# A metal tolerance protein, MTP10, is required for the calcium and magnesium homeostasis in *Arabidopsis*

**DOI:** 10.1080/15592324.2021.2025322

**Published:** 2022-01-10

**Authors:** Haiman Ge, Qiaolin Shao, Jinlin Chen, Jiahong Chen, Xueqin Li, Yu Tan, Wenzhi Lan, Lei Yang, Yuan Wang

**Affiliations:** a College of Life Sciences, Nanjing University, Nanjing, Jiangsu, China; b Cas Center for Excellence in Molecular Plant Sciences, Chinese Academy of Sciences, Shanghai, PR China

**Keywords:** MTP10, Mg homeostasis, Calcium homeostasis, *Arabidopsis*

## Abstract

Nutrient antagonism typically refers to the fact that too high a concentration of one nutrient inhibits the absorption of another nutrient. In plants, Ca^2+^ (Calcium) and Mg^2+^ (Magnesium) are the two most abundant divalent cations, which are known to have antagonistic interactions. Hence, maintaining their homeostasis is crucial for plant growth and development. In this study, we showed that MTP10 (Metal Tolerance Protein 10) is an important regulator for maintaining homeostasis of Mg and Ca in Arabidopsis. The mtp10 mutant displayed severe growth retardation in the presence of excess Mg^2+^, to which the addition of Ca^2+^ was able to rescue the phenotype of mtp10 mutant. Additionally, the deficiency of Ca^2+^ in the culture medium accelerated the high-Mg sensitivity of the mtp10 mutant. The yeast complementation assay suggested that AtMTP10 had no Ca^2+^ transport activity. And the ICP-MS data further confirmed the antagonistic relationship between Ca^2+^ and Mg^2+^, with the addition of Ca^2+^ reducing the excessive accumulation of Mg^2+^ and high-Mg inhibiting the uptake of Ca^2+^. We conclude that the Arabidopsis MTP10 is essential for the regulation of Mg and Ca homeostasis.

## Introduction

Magnesium (Mg^2+^) is essential for plant growth, while its over-accumulation in cells can be toxic. As a nutrient and signaling molecule, calcium (Ca^2+^) levels in plant cells are strictly controlled by channels, carriers, and transporters, providing a mechanistic basis for Ca^2+^ homeostasis and Ca^2+^ signaling. Both Ca^2+^ and Mg^2+^ are absorbed by plants from the soil and then translocated from the roots to the shoots through the xylem.^1^ Typically, serpentine soils show low Ca^2+^ but high Mg^2+^ concentrations, which often cause Mg^2+^ toxicity, leading to reduced crop yields.^2^ Ca^2+^ and Mg^2+^ are the two most abundant divalent cations, which appear to have antagonistic interactions in the plant cell, and the homeostasis balance between them is crucial for plant growth and development.^1^ It is widely believed that Ca^2+^ and Mg^2+^ can compete for the same enzymes or channels, transporters, etc. Notwithstanding their distinct physiological and biochemical roles, Ca^2+^ and Mg^2+^ homeostases in plants appear to be closely linked and may be regulated, at least partially, by a common signaling network. There are several ion channels and transporters that have been identified, and they are localized to the plasma membrane and various intracellular membrane systems to mediate the transport of Ca^2+^ or Mg^2+^. For example, the Ca^2+^/H^+^ exchange activity of the vacuole in the *cax1* mutant is inhibited, thereby causing Ca^2+^ to be poorly sequestered into the vacuole and leading to elevated Ca^2+^ concentrations in the cytoplasm. Consequently, the *Arabidopsis cax1* mutant is more resistant to high-Mg toxicity compared to the wild-type plants.^3^ Consistently, it has also been shown that the Mg deficiency phenotypes upon multiple knockouts of *Arabidopsis thaliana* MRS2 clade B genes, AtMRS2-1 and AtMRS2-5, can be ameliorated by concomitantly reduced Ca^2+^ supply.^4^ A Na^+^-K^+^ transporter family member OsHKT2;4 in rice is permeable to both Ca^2+^ and Mg^2+^ when heterogeneously expressed in both bacterial and oocyte systems. Moreover, the overexpression of OsHKT2;4 in the *Arabidopsis mgt6* mutant results in the seedlings being more sensitive to high-Mg stress,^5,6^ implying an interaction between Ca^2+^ and Mg^2+^. A study on *Vicia faba* guard cells suggested that Mg^2+^ can prevent continuous Ca^2+^-leakage through the channels including CGNCs, thereby assigning Mg^2+^ an important role in Ca homeostasis and Ca^2+^-dependent downstream signaling.^7^ A further example of understanding Ca^2+^-Mg^2+^ interactions come from the findings that Ca^2+^ signaling networks also function in the regulation of Mg homeostasis. In yeast, Mg^2+^ deprivation elicits rapid Ca^2+^ uptake and activates Ca^2+^/calcineurin signaling.^8^ It has been suggested that high-Mg stress is also capable of activating relevant ion channels, leading to a rapid elevation of Ca^2+^ concentration in the cytoplasm and stimulating specific Ca^2+^ oscillation.^1^ In *Arabidopsis*, two tonoplast-localized calcineurin B-like (CBL) proteins, CBL2 and CBL3, can perceive the Ca^2+^ signal and activate a subset of CBL-interacting protein kinases (CIPKs), CIPK3, 9, 23, 26, which in turn phosphorylate the as yet unknown Mg^2+^ transporters on the tonoplast, ultimately regulating Mg^2+^ sequestration into the vacuole to reduce the Mg^2+^ toxicity in the cytoplasm.^9^

In *Arabidopsis*, the MTPs (Metal tolerance proteins) belong to the CDF (cation diffusion facilitator) family, and these proteins have been identified as proton/divalent cation transporters, responsible for the efflux of cations from the cytoplasm to the extracellular space or the transport of cations from the cytoplasm to subcellular organelles, consequently making this type of transporters essential to metal tolerance.^10^ Hitherto, most of the functionally well-defined MTP transporters are capable of transporting divalent metal ions, such as Zn^2+^, Mn^2+^, Co^2+^, and Ni^2+^.^10^ Previous studies have shown that MTP10 is capable of transporting Mn^2+^ in *Arabidopsis*.^11^ However, our very recent study showed that plasma membrane-localized MTP10 can transport Mg^2+^ in the bacterial strain MM281 system, and *MTP10* loss-of-function mutant exhibits sensitivity to high-Mg stress.^12^ In this study, we further provided evidence that MTP10 is an important contributor to the regulation of Ca and Mg homeostasis. The high-Mg-sensitive phenotype of the *mtp10* mutant was effectively alleviated when the concentration of Ca^2+^ was appropriately increased in the medium. Under Ca^2+^ deficiency conditions, it is instead in a position to exacerbate the high-Mg-sensitive phenotype of the *mtp10* mutant. Thus, our study revealed that MTP10 is involved in regulating not only Mg homeostasis but also Ca homeostasis in *Arabidopsis*.

## Material and Methods

### Plant materials and growth conditions

*Arabidopsis thaliana* ecotype Col-0 was used in this study. The T-DNA insertion mutant *mtp10* (SALK_121470) was obtained from Arabidopsis Biological Resource Center (http://abrc.osu.edu). The *Arabidopsis* seeds were sterilized using 75% ethanol, and plated on 1/2 strength Murashige and Skoog (MS) medium containing 1% (w/v) sucrose. The pH of the medium was adjusted to 5.7 using KOH and solidified using 1% (w/v) agar. After 2 days of stratification at 4°C, the seedlings were then grown in a growth chamber under 150 µmol/m2/s light intensity with a 16-h light/8-h dark photoperiod at 22°C. For the phenotypic assay, the seedlings were grown in 1/2 strength MS medium for 3 days, and then transferred to a 1/6 MS containing different concentrations of MgCl_2_ or CaCl_2_ for a designed time. For the hydroponic culture, seeds of *A. thaliana* (Col-0 and *mtp10*) were grown on 1/2 strength MS for 7 days, then transferred to 1/6 strength MS solution and grown for the indicated time period.

### Quantitative RT–PCR

Seedlings of wild-type Col-0 and *mtp10* mutant were grown in 1/2 MS agar medium for 7 days and then transferred to 1/6 MS liquid medium for another 7 days. The seedlings were then treated with high-Mg (1/6 MS+10 mM MgCl_2_) for 10 h. Total RNA was isolated using Trizol reagent (Invitrogen), treated with DNase I (Invitrogen), and used as a template to synthesize first-strand cDNA with M-MLV Reverse Transcriptase (Promega) and an oligo dT primer. The qRT-PCR was performed on a Light Cycler 96 (Roche Diagnostics) with SYBR Green I Master mix (Roche Diagnostics) according to the manufacturer’s instructions. *ACTIN2* (At3g18730) was used as an internal standard. The primers are listed in Table S1.

### Yeast two-hybrid assays (Y2H)

The non-transmembrane C terminal region sequence of MTP10 was cloned into the vector pGADT7, and the CDS sequence of CIPKs was cloned into the vector pGBKT7, respectively. The fusion constructs were transformed into yeast strain AH109 using the lithium acetate transformation method as previously reported.^9^ Transformants were then grown in a synthetic dropout medium lacking tryptophan and leucine, and a synthetic dropout medium lacking tryptophan, leucine, histidine, and adenine at different dilutions (10^−1^, 10^−2^, and 10^−3^) cells/ml at 30°C.

### Bimolecular fluorescence complementation (BiFC) Assay

To generate BiFC constructs, the coding sequence of CIPK7 and CIPK26 without the stop codon was in-frame cloned into the pSPYNE(R)173-CAMBIA1300 vector, and the coding sequence of MTP10 was sub-cloned into the pSPYCE(M)-CAMBIA1300 vector.^13^ After transfection, N. benthamiana were cultured for 48 h, YFP signals were imaged by the LSM710 META confocal laser scanning microscope (Carl Zeiss). The excitation wavelength for YFP was 514 nm, and the emission wavelength was between 525 and 575 nm.

### Heterologous expression of MTP10 in yeast Δgdt1

For complementation of the yeast mutant, the full-length CDS of MTP10 was cloned into the pYES2 vector. The empty pYES2 vector and the recombinant MTP10-pYES2 plasmid were then introduced to the yeast strain *Δgdt1*, respectively. The wild-type yeast strain BY4741 was set as control. The method of the transformation of the plasmids into the yeast is described in the yeast protocols handbook (Clontech Laboratories). The cells transformed with the indicated constructs were grown on the synthetic medium containing amino acids without uracil (SC-U) and in the presence of 0, 100, 200, or 300 mM Ca^2+^.

## Results

### External Ca^2+^ can rescue the high-Mg sensitive phenotype of mtp10

**Figure 1.**
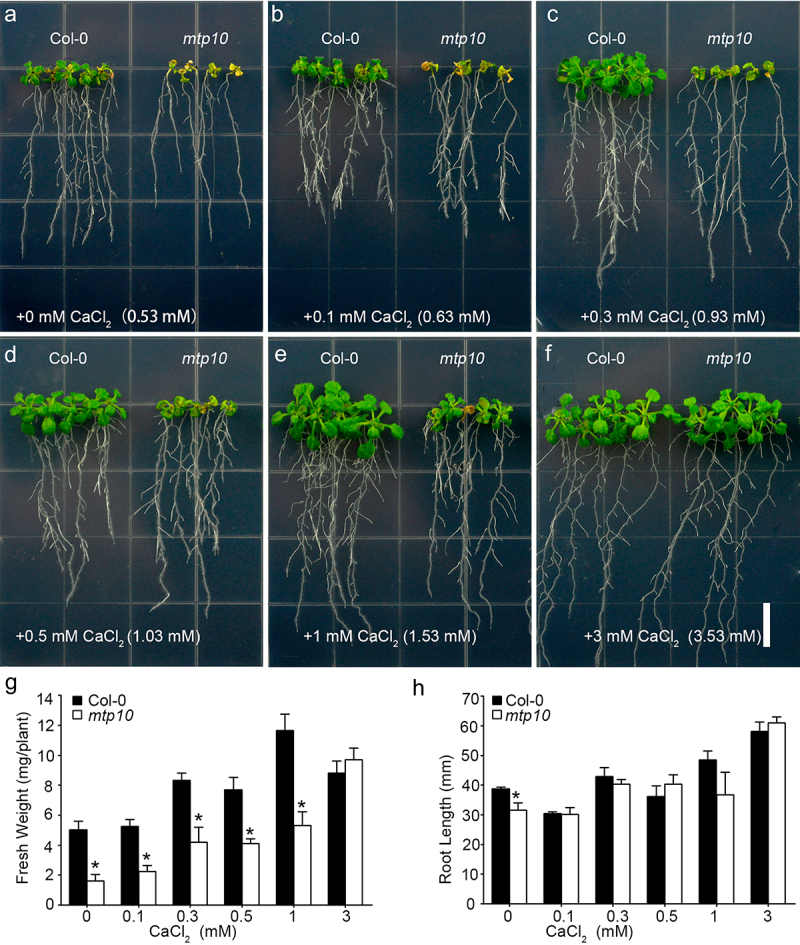
External supplementation of Ca^2+^ rescues high-Mg sensitivity of *mtp10* mutant in a dose dependent manner (a) The phenotype of Col-0 and *mtp10* mutant grown in 1/6 MS medium supplemented with 10 mM MgCl2 and different concentrations of Ca^2+^ conditions. Seedlings were grown in 1/2 MS medium for 3 days, and then transferred to 1/6 MS medium supplemented with 10 mM MgCl_2_ and external different concentrations of CaCl_2_ (0, 0.1, 0.3, 0.5, 1, and 3 mM). Bar = 1 cm. (b,c) Quantification of average primary root length (b) and fresh weight (c) of wild-type Col-0 and *mtp10* mutants. Data represent means ± SD of five replicate experiments. Asterisks indicate significant difference between the wild-type Col-0 and *mtp10* mutant (Student’s t-test, *P < .05).

We previously found a *mtp10* mutant and characterized its hypersensitive phenotype under high Mg^2+^ conditions.^12^ Based on the antagonistic relationship between Ca^2+^ and Mg^2+^, we further conducted a detailed assessment of Ca^2+^ rescue ability of *mtp10* under high Mg^2+^ conditions. The 1/6 MS medium^9^ was supplemented with 10 mM MgCl_2_ and a broad range of Ca^2+^ concentrations were used for growth assays. In the 1/6 MS medium, it had already contained 0.53 mM Ca^2+^. On the 1/6 MS medium supplemented with 10 mM MgCl_2_, the growth of *mtp10* mutant was significantly inhibited (Figure 1a). After adding 0.1 mM extra Ca^2+^ to the medium, the growth of both wild-type plants and *mtp10* mutant were recovered slightly (Figure 1b). After adding 0.3 mM, 0.5 mM, or 1 mM extra Ca^2+^ into the medium, the growth of *mtp10* mutant was significantly recovered (Figure 1c–e). Further increasing the total of Ca^2+^ to 3.53 mM in the medium can fully rescue the growth of *mtp10* mutant (figure 1f). Quantification of seedling fresh weight (Figure 1g) and primary root length (Figure 1h) further confirmed that supplementation of external Ca^2+^ can rescue high-Mg sensitive phenotype of *mtp10* mutant in a dosage-dependent manner. The results further suggested a mutually antagonistic relationship between Ca^2+^ and Mg^2+^, and also indicated that *Arabidopsis* MTP10 plays an important role in the regulation of plant Mg and Ca homeostasis.

### The mtp10 mutant is hypersensitive to high Mg^2+^ conditions in the deficiency of Ca^2+^

**Figure 2.**
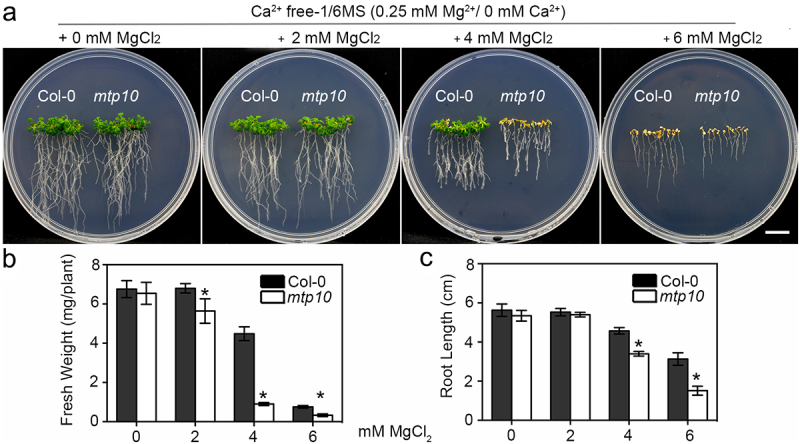
Ca^2+^ deficiency can increase the sensitivity of *mtp10* to high-Mg stress (a) The phenotype of Col-0 and *mtp10* mutant grown in Ca^2+^ free 1/6 MS medium supplemented with different concentrations of Mg^2+^. 3-day-old wild-type Col-0 and *mtp10* mutant seedlings were planted on 1/2 MS medium and then transferred to the 1/6-MS (without Ca^2+^) supplemented with different concentrations of MgCl_2_ (2, 4 and 6 mM) for another two weeks. Bar = 1 cm. (b,c) Quantification of average primary root length and fresh weight of wild-type Col-0 and *mtp10* mutants. Data represent means ± SD of five replicate experiments. Asterisks indicate significant difference between the wild-type Col-0 and *mtp10* mutant (Student’s t-test, *P < .05).

To verify the antagonistic relationship between Mg^2+^ and Ca^2+^ in plants, we performed growth assays using Ca^2+^-free 1/6 MS medium supplemented with different concentrations of Mg^2+^, in which the Ca^2+^ was removed as much as possible. After growing on Ca^2+^-free 1/6 MS medium for 2 weeks, the growth of *mtp10* mutant plants was comparable to that of wide-type plants (Figure 2). After adding extra 2 mM Mg^2+^ to the Ca^2+^-free 1/6-MS medium, the fresh weight of *mtp10* mutant was slightly lower than that in the wild-type Col-0, and the leaves of *mtp10* mutant were significantly yellower than those in the wild-type Col-0 (Figure 2a,b). The *mtp10* mutant wilted away directly under the Ca^2+^-free 1/6-MS medium supplemented with extra 4 mM MgCl_2_ (Figure 2). The growth of *mtp10* mutant was much more severe than the plants grown in 1/6 MS containing 10 mM MgCl_2_ (Figure 1a). In the presence of 10 mM MgCl_2_ supplementation in the Ca^2+^-free 1/6-MS medium, both the wild-type Col-0 and *mtp10* mutant were withered (Figure 2a). Compared to our previous data, the *mtp10* mutant only showed hypersensitive to 8–12 mM MgCl_2_ when in the 1/6 MS medium containing 0.53 mM Ca^2+^,^12^ we suggested that the deficiency of Ca^2+^ can aggravate the hypersensitivity of *mtp10* to high-Mg stress.

### MTP10 affect the accumulation of Ca^2+^ in Arabidopsis

In rice, one of the high-affinity K^+^ transport (HKT) family proteins, OsHKT2;4, was characterized a***s*** a calcium-permeable cation channel that conducts current carried by a wide range of monovalent and divalent cations.^5^ Further work also revealed that OsHKT2;4 could rescue the growth of MM281 in Mg^2+^-deficient conditions, suggesting that OsHKT2;4 can transport both Mg^2+^ and Ca^2+^.^6^ Considering that the loss-of-function of MTP10 results in seedlings being sensitive to both high-Mg and high-Ca stresses, we wondered whether MTP10 can also transport Ca^2+^. We then introduced the MTP10 in the yeast strain *∆gdt1*, which had been well used to detect the Ca^2+^ transport activity of heterogeneously expressed proteins.^14^ Under 0, 100, and 200 mM Ca^2+^ conditions, the growth of wild-type BY4741 yeast strain, the yeast mutant ∆*gdt1* expressing the pYES2 empty vector, and ∆*gdt1* expressing the pYES2-MTP10 showed no difference. Under 300 mM Ca^2+^ conditions, BY4741 yeast showed resistance to high-Ca stress, suggesting that BY4721 yeast is capable of transporting extra Ca^2+^ out of the cell, thus avoiding the toxicity of high-Ca. Both the ∆*gdt1* expressing the pYES2 and pYES2-MTP10 showed growth retardation under 300 mM Ca^2+^ condition. The growth of *∆gdt1* strains expressing pYES2 was not significantly distinguished from that of *∆gdt1* strains expressing pYES2-MTP10, indicating that MTP10 had no Ca^2+^ transport activity (Fig. S1).

**Figure 3.**
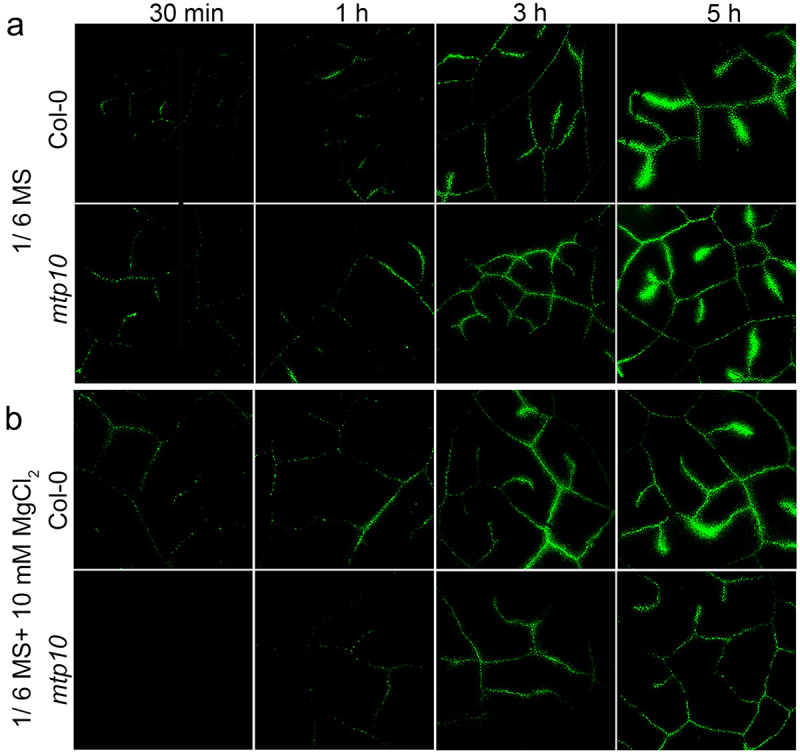
Fluorescence microscopy of extracellular Ca^2+^ in leaves of Col-0 and *mtp10* mutant. Col-0 and *mtp10* seedlings were grown on 1/2 MS solid plates for 7 days and then cultured hydroponically in 1/6 MS medium (a) or 1/6 MS containing 10 mM MgCl_2_ (b) for another 3 days. Fully expanded leaves were detached and inserted into 200-μL tubes with their corresponding growth medium in the presence of 200-μM fluorescent dye OGB-5 N. Images of the same leaf was taken at different time points (30 min, 1 h, 3 h, and 5 h) of the OGB-5 N treatment.

The Oregon Green BAPTA 488 5N (OGB-5N) is a well-established membrane impermeable low-affinity Ca^2+^ fluorescent dye that has been used to track extracellular Ca^2+^ transport and distribution in the leaf.^15^ We first grew the wild-type Col-0 and *mtp10* mutant in the 1/2 MS agar medium for 7 days, then the detached mature leaves were cultured in 1/6 MS liquid medium or 1/6 MS containing 10 mM MgCl_2_. Meanwhile, the 200 μM OGB-5 N was applied in the medium. The fluorescence signals at the time point 30 min, 1 h, 3 h, and 5 h were monitored, respectively. We found a gradually increased fluorescence signal in the vascular system over time (Figure 3). Under controlled conditions, there was no significant difference between wild-type Col-0 and *mtp10* mutant (Figure 3a). However, in the presence of 10 mM MgCl_2_ supplementation, the *mtp10* mutant showed a weaker fluorescence signal compared to the wild-type Col-0 (Figure 3b). Our results further suggest that MTP10 is involved in the homeostasis balance between Mg and Ca.

### MTP10 loss-of-function mutant showed defects in the regulation of Ca and Mg homeostasis in Arabidopsis

**Figure 4.**
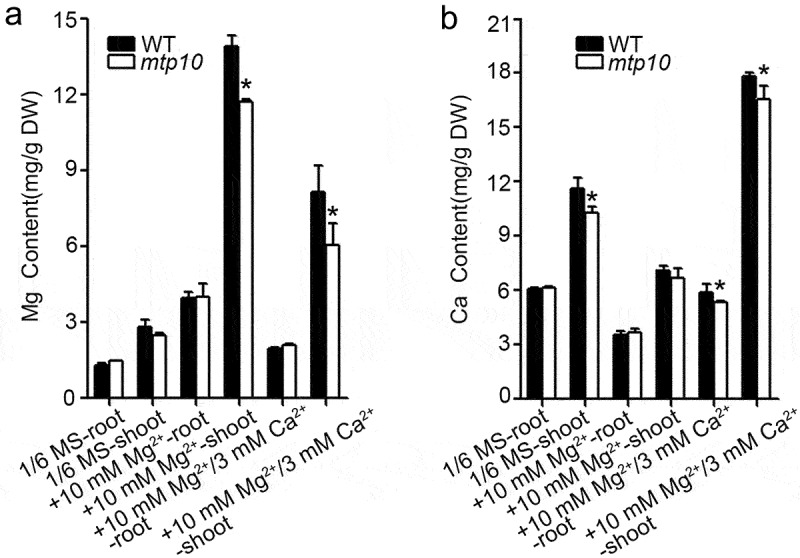
Metal ions contents in wild-type Col-0 and *mtp10* mutant. 7-day-old seedlings grown on 1/2 MS medium were transferred to 1/6 MS liquid medium, 1/6 MS supplemented with 6 mM MgCl_2_, or 1/6 MS supplemented with 6 mM MgCl_2_ and 3 mM CaCl_2_. Samples were collected after being treated for another 10 days. The Mg (a) and Ca (b) contents were measured by ICP-MS. Data are means ± SD. n = 4. Asterisks indicate significant difference between the wild-type Col-0 and *mtp10* mutant (Student’s t-test, *P < .05).

To investigate the causes of Ca^2+^ being able to rescue the high-Mg sensitive phenotype of *mtp10*, we measured the major cation contents in wild-type Col-0 and *mtp10* mutant under different treatment conditions by using the ICP-MS. The 7-day-old seedlings grown on 1/2 MS medium were transferred to 1/6 MS liquid medium, 1/6 MS supplemented with 6 mM MgCl_2_, or 1/6 MS supplemented with 6 mM MgCl_2_ and 3 mM CaCl_2_ for another 10 days. We separately sampled the roots and shoots of seedlings. Compared with the control group (1/6 MS), we found that both roots and shoots of the wild-type Col-0 and *mtp10* mutant seedlings accumulated a large amount of Mg after high-Mg treatment (1/6 MS+10 mM MgCl_2_), while the shoots accumulated more Mg than that in the roots. When plants were grown in 1/6 MS medium supplemented with 6 mM MgCl_2_ and 3 mM CaCl_2_, the seedlings accumulated a relatively lower amount of Mg compared to the treatment with only 10 mM extra MgCl_2_ (Figure 4a). Meanwhile, the shoot of the *mtp10* mutant accumulated a relatively lower amount of Mg compared to that in the wild-type Col-0 when plants were grown in 1/6 MS supplemented with 10 mM MgCl_2_ or 1/6 MS supplemented with 10 mM MgCl_2_ and 3 mM CaCl_2_ (Figure 4a). These results further suggested that high-Mg stress could lead to excessive accumulation of Mg in plants and that the addition of Ca^2+^ can suppress the over-accumulation of Mg. We also measured the Ca contents in each treatment. Under controlled conditions, the Ca content in the shoot of *mtp10* mutant was slightly lower than that in the wild-type Col-0. We also found that high-Mg treatment (1/6 MS+ 10 mM MgCl_2_) inhibited the Ca accumulation in both the roots and shoots of wild-type Col-0 and *mtp10* mutant. When plants were grown on 1/6 MS medium supplemented with 6 mM MgCl_2_ and 3 mM CaCl_2_, the shoots of wild-type Col-0 and *mtp10* mutant accumulated much higher Ca compared to other treatment groups. Moreover, the Ca contents in the root and shoot of the *mtp10* mutant were slightly lower than that in the wild-type Col-0 (Figure 4b). Together with these results, we further provide compelling evidence that a mutually antagonistic relationship exists between Ca and Mg, and that MTP10 plays an essential role in maintaining Ca and Mg homeostasis.

We also detected the expression of Mg^2+^ transport-related proteins in wild-type Col-0 and *mtp10* mutant under control conditions, high-Mg, and Mg-deficient conditions, we cultured wild-type Col-0 and *mtp10* mutant in 1/6 MS hydroponic solution for 1 week, and then transferred them to control (1/6 MS), high-Mg (1/6 MS+ 10 mM MgCl_2_), or Mg-deficient (1/6 MS-Mg^2+^) hydroponic solutions. The total RNA of each group of samples was extracted after 10 hours of treatment in the medium, and then the expression of the target genes was detected. CIPK3/9/23/26, CBL2/3, MGT2, and MHX were mentioned previously as transporters associated with Mg^2+^ transport on vesicles. MGT9 was associated with reproductive development in *Arabidopsis*, MGT10 was localized in chloroplasts, and MGT6 was associated with Mg^2+^ uptake in roots under low-Mg environment and resistance to high-Mg stress.^9^ The expression of these genes was indeed up- or down-regulated in the *mtp10* mutant compared to the wild-type Col-0, indicating the disruption of Mg homeostasis in the *mtp10* mutant (Figure S1). Under control conditions (1/6 MS), the expression of all our selected genes showed upregulation in the *mtp10* mutant, except for the expression of a downregulated MGT9. Among these genes, the *CIPK26* and *MGT10* exhibited at least a two-fold up-regulated expression in the *mtp10* mutant. When seedlings were treated with high-Mg (1/6 MS+ 10 mM MgCl_2_), all of these genes showed up-regulated expression in the wild -type Col-0 seedlings compared to the control. However, in the *mtp10* mutant, the expression patterns of these genes were differentiated. The expression of *CIPK3* and *CBL2* showed no difference between wild-type Col-0 and *mtp10* mutant. The expression of *CIPK9, CIPK23, CBL3*, and *MGT9* showed higher expression in the *mtp10* mutant compared to that in the wild-type Col-0 seedlings. While the expression of *CIPK26, MGT2, MHX, MGT6*, and *MGT10* was compromised in the *mtp10* mutant (Fig S2). Our qRT-PCR results indicated at the molecular level that Ca^2+^ signaling is involved in regulating plant responses to high-Mg stress and that MTP10 plays a role in Ca^2+^ signaling regulated adaptation to high-Mg stress.

## Discussion

Ca and Mg are secondary phytonutrients compared to nitrogen (N), phosphorus (P), and potassium (K), but are equally essential for plant growth, although the requirements of those elements are comparatively negligible to that of macronutrients. In plant cells, Ca^2+^ plays irreplaceable roles in increasing cell wall strength, neutralizing the anion potential in the vacuole, and counteracting external stresses. Ca in the form of calcium pectinate forms an intermediate gelatinous layer of the plant cell wall, enabling cells and cells to join together to form tissues and giving the plant organ or individuals a certain mechanical strength.^16^ Mg^2+^ is a component of the chlorophyll molecule, among other functions in plants, and therefore essential for photosynthesis. Ca and Mg exhibit similar chemical properties, and both are doubly positively charged in the soil-water phase and at the soil cation exchange sites.^17^ The importance of Mg and Ca lies not only in the role they play in plant growth but also in their interaction with each other. In Citrus sinensis (L.) Osbeck seedlings, Mg-deficiency induced leaf vein lignification, enlargement, and cracking.^18^ These findings imply that Ca^2+^ and Mg^2+^ may have mutual antagonism in the cell wall, affecting plant growth, development, and coping with environmental stresses. During our study, we found that the mutation of MTP10 resulted in the plants could not efficiently facilitate Mg^2+^ diffusion from the xylem to shoots.^12^ The leaf veins in the *mtp10 mgt 6* double mutant also showed abnormal morphology, suggesting that MTP10 is involved in vascular development.^12^ Under high Mg^2+^ conditions, we found that the Ca^2+^ accumulation in the vascular region of the leaf of *mtp10* mutant was inhibited (Figure 3). The ICP-MS data also suggested that high-Mg stress inhibited the accumulation of Ca in the whole plant (Figure 4b). We speculated that one of the possible reasons is that high-Mg stress affects Ca^2+^ transport in plants, especially in vascular tissues, which in turn affects the cross-linking of plant cell walls. The damage to the cell wall eventually induced high-Mg toxicity in plants, which led to the *mtp10* mutant being more sensitive to high-Mg toxicity. In conditions of Ca^2+^ deficiency, the high-Mg stress further deteriorated Ca^2+^ transport and absorption by plants, and thus the *mtp10* mutant growth was even more inhibited under low-Ca and high-Mg superposition conditions. Applying the appropriate amount of Ca^2+^ in the medium can effectively alleviate the excessive accumulation of Mg^2+^ in plants (Figure 4a), and thus the phenotype of *mtp10* mutant can be restored (Figure 1). The absence of MTP10 prevented plants from effectively regulating the balance of Ca and Mg.

In *Arabidopsis*, there are total of 12 MTP genes which had been identified and clustered in the CDF family, and several of which have been found function in metal ions transport and tolerance.^19^ However, only MTP10 was found to be localized to the plasma membrane of parenchyma cells in vascular bundles and function in unloading of xylem Mg^2+^ to confer tolerance to high-Mg stress.^12^ Among these MTP proteins, MTP1 was discovered to be involved in the response to high-Zn stress.^19^ The MTP11 was found to transport Mn^2+^ in a proton-antiport manner and thus conferring high-Mn tolerance.^20^ The tonoplast localized MTP8 was capable of coordinating Mn homeostasis and Fe reallocation.^21^ Further study suggested that calcium-dependent protein kinases, CPK4/5/6/11, can interact with and function upstream of MTP8, indicating the presence of tonoplast-associated calcium signaling cascade that orchestrates Mn homeostasis.^22^ Many studies have shown that Ca^2+^ signaling plays important role in plant response to high-Mg stress. By performing the Y2H and BiFC assay, we found that CIPK7 and CIPK26 can interact with MTP10 (Fig. S3). In *Arabidopsis*, its genome contains 34 CPKs, which are essential to growth and development, and function in response diverse to biotic and abiotic stresses.^23^ It is likely that certain CBL-CIPK complex and CPK kinases interact with MTP10 and act as upstream elements to mediate phosphorylation of MTP10 and act as a calcium signaling network in response to high-Mg stress. Besides MTP10, the CorA-type plasma membrane localized Mg^2+^ transporter MGT6 was also found to play an essential role in high-Mg tolerance.^24^ It is also possible that both the CBL-CIPK and CPKs Ca^2+^ signaling network regulate the activity of MGT6 and thus MTP10 and MGT6 synergistically regulate Mg^2+^ transport and homeostasis in *Arabidopsis*.

Apart from the interaction between Ca^2+^ and Mg^2+^, Ca^2+^ or Mg^2+^ can also affect the homeostasis of other ions, such K^+^, and Na^+^. The mechanisms of interaction between these ions are also intriguing to explore. For example, high-Mg stress can disrupt the K^+^ homeostasis, and the loss-of-function of two K^+^ transporters, AKT1 and HAK5, results in the seedlings hypersensitive to high-Mg stress.^25^ High-Na stress can interfere with K^+^ metabolism, and the presence of Ca^2+^ or Mg^2+^ reduces this interference.^26^ And if the K^+^ concentration in the cultured medium gets too high, the Ca^2+^ and Mg^2+^ uptake can be inhibited. Therefore, combining these studies and our research, we believe that understanding the relationship between ion interactions and the molecular mechanisms that mediate this relationship is a fundamental effort to achieve efficient nutrient uptake by plants and enhance crop yields.

## Supplementary Material

Supplemental Material
