# Supplementary material for: A metal tolerance protein, MTP10, is required for the calcium and magnesium homeostasis in Arabidopsis
Source: Plant Signal Behav. 2022 Jan 10;17(1):2025322. doi: 10.1080/15592324.2021.2025322 (PMC9176222; doi:10.1080/15592324.2021.2025322)
Supplement: Supplemental Material [file KPSB_A_2025322_SM5560.docx]

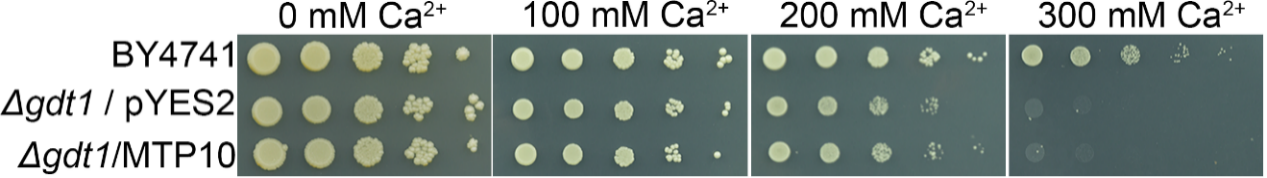


**Figure S1. *Arabidopsis thaliana* MTP10 cannot mediate Ca^2+^ transport in heterologous yeast expression system *Δgdt1*.** Complementation assay of Ca^2+^ sensitivity of *Δgdt1* yeast by AtMTP10. Serial 10-fold dilutions were dropped onto medium supplemented with CaCl_2_. The concentration of Ca^2+^ was indicated on the top. 5 μl (OD600 = 0.2) of 10-fold serial dilutions were spotted. Plates were incubated at 30 ^o^C for 3–4 days.


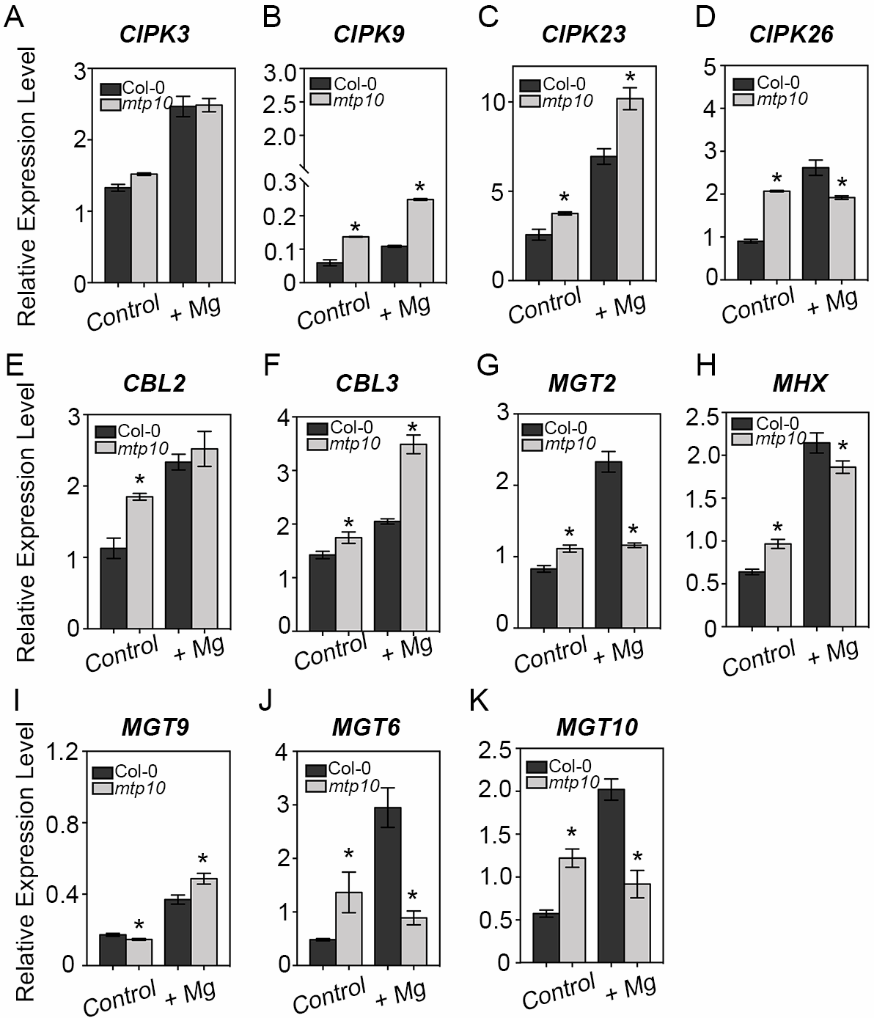


**Figure S2. Expression of Mg^2+^ tolerance related genes in Col-0 and *mtp10* mutants.** Seedlings of wild type Col-0 and *mtp10* mutant were grown in 1/2 MS agar medium for 7 days and then transferred to 1/6 MS liquid medium for another 7 days. Then the seedlings were treated with high-Mg (1/6 MS+10 mM MgCl_2_) for 10 h. The relative expression of each gene was double-normalized using the expression level of *ACTIN2* and the expression level of each gene at 0 h. Data represent mean ± SD (n = 4). Asterisks indicate significant difference between the wild type Col-0 and *mtp10* mutant (Student’s t-test, *P < 0.05).


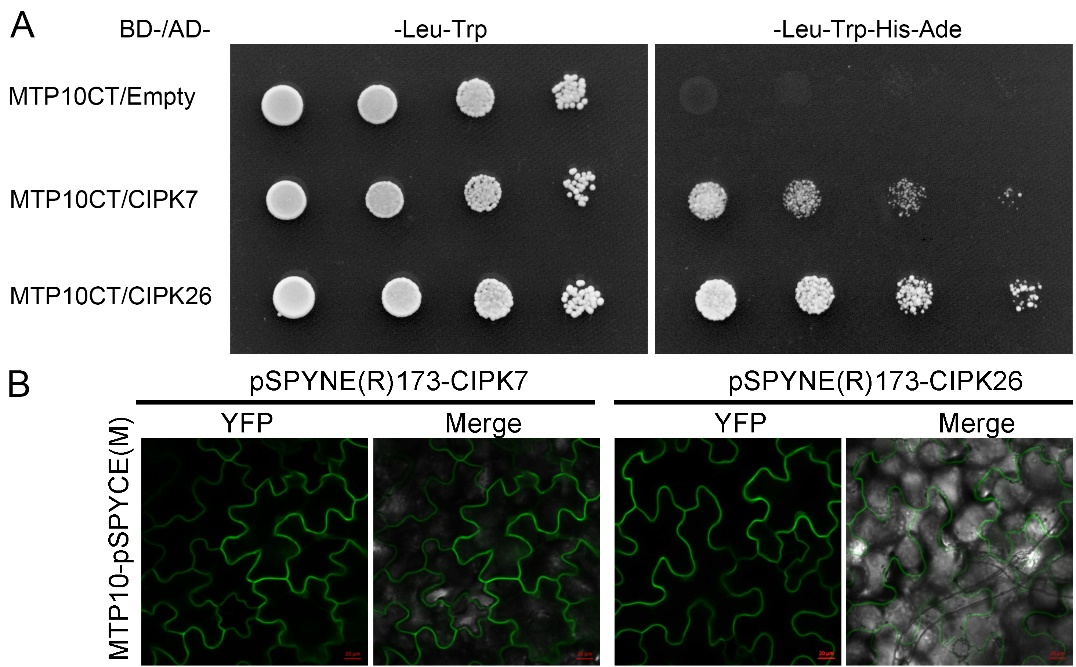


**Figure S3. CIPK7 and CIPK26 interact with MTP10.** (A) Yeast two-hybrid assay of the interactions between CIPK7/26 and MTP10. Yeast AH109 cells were transformed with various combinations of BD- and AD-fusion constructs as indicated. (B) The interaction of CIPK7/26 and MTP10 in N. benthamiana leaves were performed using BiFC assays.

**Supplemental Table 1. List of PCR Primers**

| Names | Primer sequences | Purposes |
| --- | --- | --- |
| MTP10-F | GGGGGATCCATGCCGCTTAACTCCTATATTTTCTTTC | Mutant identification of *mtp10* |
| MTP10-R | GGCTGCAGCTAGTTACACTTGTGTTCAGGACGATGAG |  |
| SKLBa1 | TGGTTCACGTAGTGGGCCATCG | Mutant identification |
| *cipk26*-F | TTGTTTTCTTCAGTCGAACCCCAAC | Mutant identification of *cipk26* |
| *cipk26*-R | TATTTGCTTAGACCAGAGCTCTCGC |  |
| \| *cipk7*-F \| TAACTCCACTGCTTCCACCAC \| \| --- \| --- \| | TAACTCCACTGCTTCCACCAC | Mutant identification of *cipk7* |
| \| *cipk7*-R \| TCCATTTAGCTCGATCAATCG \| \| --- \| --- \| | TCCATTTAGCTCGATCAATCG |  |
| CIPK7-BiFC-F | GGGGGATCCATGGAATCACTTCCCCAGCCG | BIFC |
| CIPK7-BiFC- R | GGGGGTACCCATGATGTCATTGTGCCATGAAAGAAC |  |
| MTP10-BD-F | GCGTCGACCTATGCCGCTTAACTCCTATATTTTCTTTC | Y2H |
| MTP10-BD-R | AACTGCAGCTAGTTACACTTGTGTTCAGGACGATGAG |  |
| CIPK7-AD-F | CGGAATTCATGGAATCACTTCCCCAGCCG | Y2H |
| CIPK7-AD-R | CGGGATCCTTACATGATGTCATTGTGCCATGAAAG |  |
| CIPK26-AD-F | GCGTCGACCTATGAATCGGCCAAAGGTTCAGC | Y2H |
| CIPK26-AD- R | AACTGCAGTTATTTGCTTAGACCAGAGCTCTCGC |  |
| CIPK26-BiFC-F | CGGGGTACCATGAATCGGCCAAAGGTTCAG | BIFC |
| CIPK26-BiFC-R | TCCCCCGGGTTTGCTTAGACCAGAGCTCTCGC |  |
